# Assessing the Quality of Maternal Mental Illness Care Across Maternity Care Settings in Southwestern Uganda

**DOI:** 10.7759/cureus.76670

**Published:** 2024-12-31

**Authors:** Gladys Nakidde, John F Mugisha, Edward Kumakech

**Affiliations:** 1 Reproductive Health Science, Pan African University, Life and Earth Sciences Institute (Including Health and Agriculture), Ibadan, NGA; 2 Nursing, Bishop Stuart University, Mbarara, UGA; 3 Public Health, Bishop Stuart University, Mbarara, UGA; 4 Nursing and Midwifery, Lira University, Lira, UGA

**Keywords:** assessment of chronic illness care tool, maternal mental health problems, mental health screening in maternity settings, perinatal mental illness, quality of care, women’s mental health care

## Abstract

Objective: Maternal mental illness (MMI) is associated with many adverse effects on the mother, baby, and family, which can be overcome by timely and appropriate interventions to meet women's needs. The quality of care for MMI is crucial for timely management yet not well studied, especially in low- and middle-income countries like Uganda. This study aimed to evaluate the capacity of maternity care facilities to deliver quality care for MMI and to explore the women's needs and barriers to MMI care delivery in Southwestern Uganda.

Methods: We used mixed methods to study health workers in maternity care settings as well as pregnant and postpartum women from various health facility levels. The Assessment of Chronic Illness Care (ACIC) tool was used to assess the capacity to screen and manage MMI in maternity health care settings. Antepartum and postpartum women who had been screened positive for MMI had their file records reviewed to confirm whether the health workers also detected MMI symptoms and/or documented any interventions toward their MMI symptoms. They also reported on the quality of care received and how accessible it was using both qualitative and quantitative approaches. IBM SPSS Statistics for Windows, Version 26 (Released 2019; IBM Corp., Armonk, New York, United States) was used for the analysis of quantitative findings while qualitative results followed thematic content analysis.

Results: All healthcare facilities had ACIC domain scores indicating no or little capacity to manage women with MMI. Maternal care providers detected only 33.3% (16 of 48) of mothers with MMI. A limited proportion of women with MMI received proper treatment (27.1% psychological, 8.3% pharmacological). Emerging themes from interviews included a shortage of maternity care workers as well as poor communication, assessment, and management skills among care providers. More than half (52.1%) of the women who screened positive for MMI did not consider themselves sick, and more than half (54.2%) lacked the financial means to pay for medical care services.

Conclusion: The capacity to screen and manage MMI is suboptimal, and women with MMI are unable to recognize that they are unwell. It is crucial to enhance the proportion of women with MMI who are adequately diagnosed and treated by increasing the number of maternal care providers who are skilled and knowledgeable about MMI. There is also a need to raise public awareness about the causes and presentation of MMI, as well as what constitutes quality care, to enhance health-seeking behaviors and encourage service-user-driven quality improvement.

## Introduction

Maternal mental illness (MMI), including depressive and/or anxiety symptoms, is a recent part of the significant public health concerns with a pooled global prevalence of 11.9%; moreover, it is reported to be higher in the low- and middle-income countries (LMICs) than in high-income countries (HICs) [1]. In LMICs, anxiety prevalence during the perinatal period is said to range from 1% to 26%, while perinatal depression ranges from 1% to 37% [2]. MMI presents with adverse effects including loss of productivity, intrauterine growth restriction, low birth weight, prematurity, poor mother-child bonding in the short term, infanticide, maternal suicide, and abnormal behavioral, emotional, and cognitive development in children, among others, in the long run [3,4]. Consequently, MMI contributes significantly to maternal and infant morbidity and mortality and hence is a key barrier to the achievement of the sustainable development goal (SDG) number three (3), which focuses on the reduction of maternal morbidity and mortality by the year 2030 [5] and the African Union’s Agenda 2063 that emphasizes healthy citizenry and high-quality life [6].

To enhance the attainment of SDGs related to maternal and infant health, numerous programs and projects have been rolled out by WHO under the Mental Health Gap Action Program (mhGAP) to raise the capacity of non-specialized healthcare workers to detect and manage mental problems at primary and tertiary health care facilities in LMICs over the last 10-20 years [7]. Some of the projects include the “friendship bench” in Zimbabwe [8], the “stepped up project” in South Africa [9], and the Program for Improving Mental health Care (PRIME) project in Uganda, Nepal, Ethiopia, South Africa, and India [10], which have helped to evaluate the impact of integrating mental health care in primary health care. They have consequently reported many benefits, such as increased knowledge, improved competencies like communication skills and empathy, as well as a significant reduction in symptom severity and functional impairments associated with MMI [11]. However, these studies also pointed out a number of key care provider competencies that were either impacted marginally or not at all, such as the ability to assess and detect mental illness, involving family members in care, and assessing critical symptoms like suicide risk and barriers to the integration of mental health care in PHC, including limited funding, insufficient specialists to supervise non-specialist workers, inadequate health system structures to support the rollout of task-shared interventions, low community awareness of mental health, and high levels of stigma [7,11,12]. Very few studies have reported on the quality of mental health care specifically in perinatal care settings following the implementation of interventions to enable non-specialist health care workers to assess and manage mental illness.

It is worth noting that the quality of care is one of the most critical requirements among the strategies for enhancing maternal and infant health in general [13]. According to Hulton et al., quality of care “is the degree to which maternal health services for individuals and populations increase the likelihood of timely and appropriate treatment for the purpose of achieving desired outcomes that are both consistent with current professional knowledge and uphold basic reproductive rights” [14]. Given that what the care providers may consider to be high-quality care may not be acceptable to the care recipients and vice versa, the Hulton et al. definition suggests that the quality of a maternal care service should be assessed from two perspectives: the care provided and the woman's experiences with that care [14,15]. The quality of care given to mothers and newborns in many LMICs is poor, resulting in low detection rates of MMI and delayed or no interventions for symptomatic women [16].

In Uganda, information about the quality of care provided and received by MMI symptomatic mothers is not well studied and published. Therefore, this study had four major objectives: 1) to evaluate the capacity of maternity care facilities to deliver MMI care; 2) to assess the ability of maternal care providers to identify and treat MMI; 3) to assess the quality of care received and reported by MMI symptomatic women; and 4) to explore women's mental health needs as well as barriers to MMI care delivery and access in Southwestern Uganda. The study's findings would help policymakers and healthcare providers enhance maternal mental health care.

## Materials and methods

Study design

This is part of a larger study on maternal mental health care in southwestern Uganda. This sub-study was mixed and cross-sectional in nature, employing both qualitative and quantitative methods.

Study setting

The study was conducted in the antenatal and postnatal settings in health centers at different levels in Mbarara and Kabale districts, southwestern Uganda, a rural region [17]. The two districts were purposefully chosen because they have a large number of health facilities and are home to the only two regional referral hospitals in the whole Southwestern region, resulting in a larger catchment population for representation [17].

The Ugandan health care system is organized according to different levels (Health Center I, II, III, IV, and referral level), and the usual criteria are based on the level of training of health workers, the kind of services offered, and the target population [18]. Health Center I (HCI) is at the village level and comprises community health workers also known as village health teams (VHTs). VHTs are village volunteers who do not have a physical office and mostly work on community-based preventive, health awareness, and basic curative activities. HCII (parish level) is staffed by primary health care providers (e.g., general nurses or midwives) who provide basic health services. HCIII (sub-county level) health centers have a clinical officer, a maternity center, and, unlike the lower levels, distinct wards for male and female patients (eight beds). Maternity health services begin at HCIII, with referrals to HCIV at the county level (district hospitals), regional referral hospitals, and, finally, national referral hospitals [17].

Study population and inclusion criteria

All women of reproductive age who were pregnant (antepartum) or were within one year after delivery (postpartum) and screened positive for depression or anxiety using the Edinburgh Postnatal Depression Scale at the time of data collection were included in the study. Women with pre-existing chronic MMI and those receiving known treatment for mental health disorders were excluded. We also included all healthcare providers who provided maternity care to women at participating healthcare facilities, such as midwives, nurses, doctors, obstetricians, and mental clinical officers.

Sample size and sampling procedure

Multistage cluster sampling was done to select the 20 health facilities included in the study. The two referral hospitals and six HCIV facilities were all included in the study. Random sampling was used to select 12 HCIII facilities from a combined total of 24 HCIII units in the two districts. Health workers in the postnatal and antenatal departments were selected by purposive sampling to ensure representation from various health facility levels. The women who were screened were selected by consecutive sampling from the respective health facilities, while the qualitative sample was purposively selected. The sample size of women who were eligible to participate in the study was 72, and this included women who screened positive for depression and/or anxiety on the Edinburgh Postnatal Depression Scale for depression and anxiety who returned for the interview after assessment by the resident health care worker at the facility. The sample size of maternal care providers who participated was 38. It was determined using the Yamane formula [19]: \[ n = \frac{N}{1 + N e^2} \]where n is the desired sample size, N=40, the population of expected health workers (at most two from each facility, considering frontline health care workers and/or those in management positions) from the 20 health facilities visited for data collection in Kabale and Mbarara, and e is the level of precision, which equals 0.05.

Data collection tools and procedures

**Figure 1:**
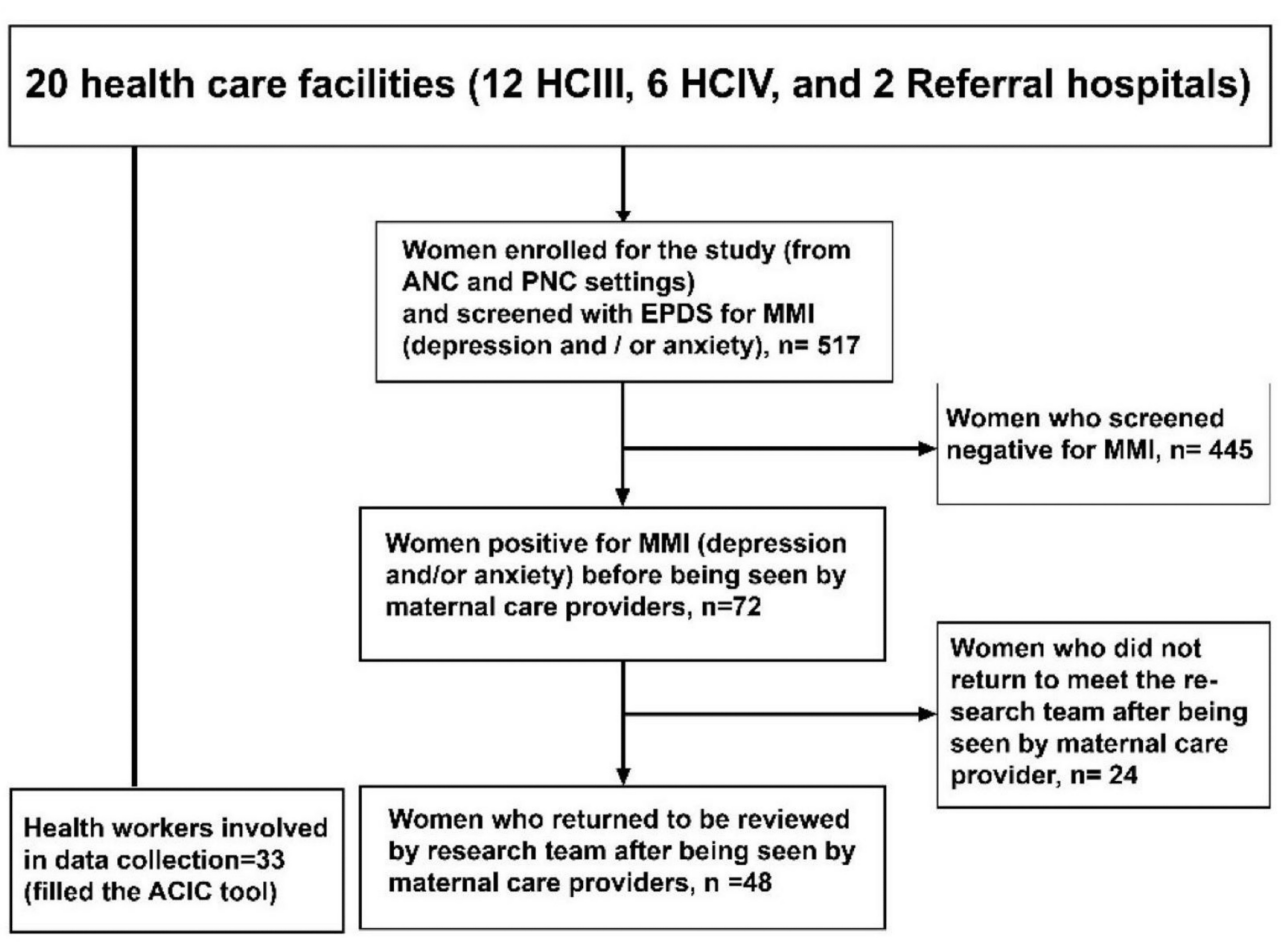
Participant recruitment flow chart HCIII: Health Center III; HCIV: Health Center IV; ANC: antenatal care; PNC: postnatal care; EPDS: Edinburgh Postnatal Depression Scale; MMI: maternal mental illness (depression and/or anxiety); ACIC: Assessment of Chronic Illness Care

In addressing the study objectives, we used a mixed-methods approach. For the first study objective, we administered a questionnaire to health workers to assess the capacity of maternal care providers and facilities to manage MMI (Appendices A-B), which had two sections: A for health workers' sociodemographic characteristics (Appendix A) and B for assessing MMI care in maternity care settings on the ACIC tool components (Appendix B). The ACIC is a tool used widely to evaluate the standard of care given to patients who require chronic care [16,20]. The ACIC tool has 34 items spread across seven domains, namely: i) organization of the healthcare delivery system (six items), ii) community linkages (three items), iii) patient support for self-management (four items), iv) decision support for service providers (three items), v) delivery system design (six items), vi) clinical information system (five items), and vii) integration of chronic care components (six items). Each item in the individual domains was scored on a four-point Likert scale, giving an average domain score ranging from 0 to 11. A score between “0” and “2” corresponded to the lowest level of support for MMI care (little or no support for MMH illness care); “3” and “5” corresponded to basic support (basic or intermediate support for MMH care), and “6” and “8” corresponded to reasonably good support, while between “9” and “11” indicated fully developed MMH care programs (optimal or comprehensive integrated MMH care) [20]. Maternal care providers scored the individual components of the ACIC tool according to how they viewed the performance of maternal mental care services in their settings. The scores for the seven components were obtained by obtaining total scores for the individual parameters under that component divided by their number of items, and the overall average was obtained by the sum of the scores for the seven components divided by the total number of the items. To address the second, third, and fourth objectives, we administered a questionnaire (Appendices C-E) to women who had been screened positive for MMIs (depression and anxiety), which was developed based on literature [21]. We tested the internal consistency of the questionnaire, which was very good at Cronbach’s Alpha 0.833 and interclass correlational coefficient 0.840 (95% Cl 0.769-0.899). We assessed the ability of maternal care providers to detect and treat MMI (Table 7) as well as the level of satisfaction and perceived accessibility of MMI care by women with symptomatic MMI (Table 8). We consecutively recruited and screened women who came for routine perinatal care and had voluntarily consented to participate in the study for perinatal mental illness using the Edinburgh Post-natal Depression Scale (EPDS) to identify those with perinatal depression and/or anxiety before they had been seen by the maternal care providers. The participant recruitment process is summarized in Figure 1.

The EPDS is a validated screening tool in our local setting (Uganda), and for the purpose of this research study, we used a cut-off score of ≥10 out of a possible total of 30 to indicate depression [22]. The three items in the EPDS (questions 3, 4, and 5) comprise the EPDS anxiety subscale (EPDS-3A), and a score of 5 and above is indicative of anxiety [23]. After being reviewed by the maternal care providers, we then determined whether a participant who had been diagnosed with depression and/or anxiety using the EPDS before they had been seen by the maternal care providers had been recognized as having a psychological problem by the maternal care provider by meticulously reviewing the case records made on each of the participants by the care providers to see whether any entry had been made to indicate that a psychological or emotional problem or diagnosis had been noted or treated. Any participants whose case records did not show any entry of possible psychological problems were deemed an undetected case of MMI.

To address objective 4, i.e., we qualitatively interviewed women using open-ended questions in line with the study objective (Section C) to explore women's expectations in care and barriers to MMI care delivery and access in a rural setting. Each interview was conducted until no new information was generated during the succeeding interview. The lead author and three trained research assistants collected data between July and September 2022, conducted in the local language (Runyankore-Rukiga), and they were audio recorded and supplemented with field notes with the participants' consent. One team member led the interview, while the other audio recorded and took notes. The members switched roles during subsequent interviews.

Data management and analysis

Quantitative Data

The data from the questionnaires was entered, coded, cleaned, and analyzed using IBM SPSS Statistics for Windows, Version 26 (Released 2019; IBM Corp., Armonk, New York, United States). Assessment of the capacity of health workers and the system to manage MMI from the ACIC tool was reported in proportions at various levels of support. An average for the individual domain scores was obtained by dividing the total score for the domain by the number of items in that domain category. The data on the detection and management of MMI by healthcare providers and patients’ reports from case records were presented in proportions. Chi-square or Fischer’s exact test was reported for the association between interventions by health care providers and the women’s rating of care and accessibility by health care providers; p-values at statistical significance of less than 0.05 were reported.

Qualitative Data

The qualitative data was analyzed using thematic content analysis. Data from in-depth interviews was transcribed verbatim, cleaned, and cross-checked against audio recordings to ensure accuracy. Each of the authors independently reviewed the processed data for coding and analysis. The authors collaborated to develop codes based on the broad themes identified in the data. The authors independently developed codes based on emerging themes from the data and then discussed them as a team. The team created a codebook, presented it, and discussed it before finalizing it for use in analysis. The first author then went through all of the transcripts several times and assigned codes to each piece of information gathered. Data matrices were created from the data sets and then organized into broad themes that reflected the study's objectives. Emerging patterns in the data were explained using rich verbatim quotes from participants to highlight key issues within the themes.

Ethical considerations

Ethical approval from the University of Ibadan Ethical Review Board (approval no.: 22/0018), Mbarara University of Science and Technology Research Institutional Review Board, Uganda (approval no.: MUST-2022-414), and the Uganda National Council for Science and Technology (approval no.: SS1362ES). Every participant gave verbal and written consent before taking part in the study. Confidentiality was guaranteed by assigning non-identifiable field codes to each participant and conducting the interviews in a secluded area. We respected participants' right to decide whether or not to participate in the study and excluded those who declined. All who agreed to participate were advised that they might withdraw from the study at any moment without penalty.

## Results

We obtained qualitative and quantitative findings from 33 health workers (87% response rate) and 48 perinatal women who were screened positive for depression and anxiety during pregnancy and after delivery (67% response rate). The low response rate was due to the fact that not all women screened positive for MMI returned for an interview after being reviewed by the health workers. We assessed the health workers’ capacity to screen and manage mental problems in the maternity care settings using the ACIC tool and explored whether health workers detected MMI symptoms and documented possible management interventions for MMH problems. This was obtained from a review of perinatal women’s files or documents following health worker assessment and documentation. We further obtained perinatal women’s reports on whether the available services meet their needs and barriers to MMI care.

Participants’ characteristics

We obtained participants' sociodemographic characteristics as shown in Table 1 below.

**Table 1:** Sociodemographic characteristics of health workers and women who screened positive for maternal mental health problems sd: standard deviation

Health workers characteristics	
Variable, n=33	Frequency (percentage)
Gender of health workers	
Male	6 (18.2)
Female	27 (81.8)
Mean age in years (sd)	36.4 (6.59)
Median age in years (range)	35 (28-52)
Health facility level	
Health Center III	16 (48.5)
Health Center IV	10 (30.3)
Referral hospital	7 (21.2)
Position held by staff	
Unit In-charge	19 (57.6)
Frontline healthcare provider	14 (42.4)
Department, n=32	
Antenatal	20 (60.6)
Postnatal	11 (33.3)
Young child clinic	1 (3.0)
Designation	
Midwife	22 (66.7)
Nurse	6 (18.2)
Doctor	1 (3.0)
Others	4 (12.1)
Health worker's qualifications	
Degree	2 (6.1)
Diploma	21 (63.6)
Certificate	10 (30.3)
Mean years of experience (sd)	11.79 (5.67)
Median years of experience (range)	10 (2-25)
Characteristics for women who screened positive	
Health facility level	
Health Center III	17 (35.4)
Health Center IV	14 (29.2)
Referral hospital	17 (35.4)
Department	
Antenatal	28 (58.3)
Postnatal	14 (29.2)
Young child clinic	6 (12.5)

From Table 1, most health workers were females (81.6%) and diploma holders (63.8%). Midwives were the majority (66.7%) since data collection was done in maternity care settings where midwives predominate, and midwifery training in Uganda only allows females to enroll for the program at the certificate and diploma levels. The majority of MMI problems were reported during the antenatal period (58.3%) rather than in the postpartum period.

Health workers’ rating of health care quality for MMH problems using the Assessment of Chronic Illness Care tool

**Figure 2:**
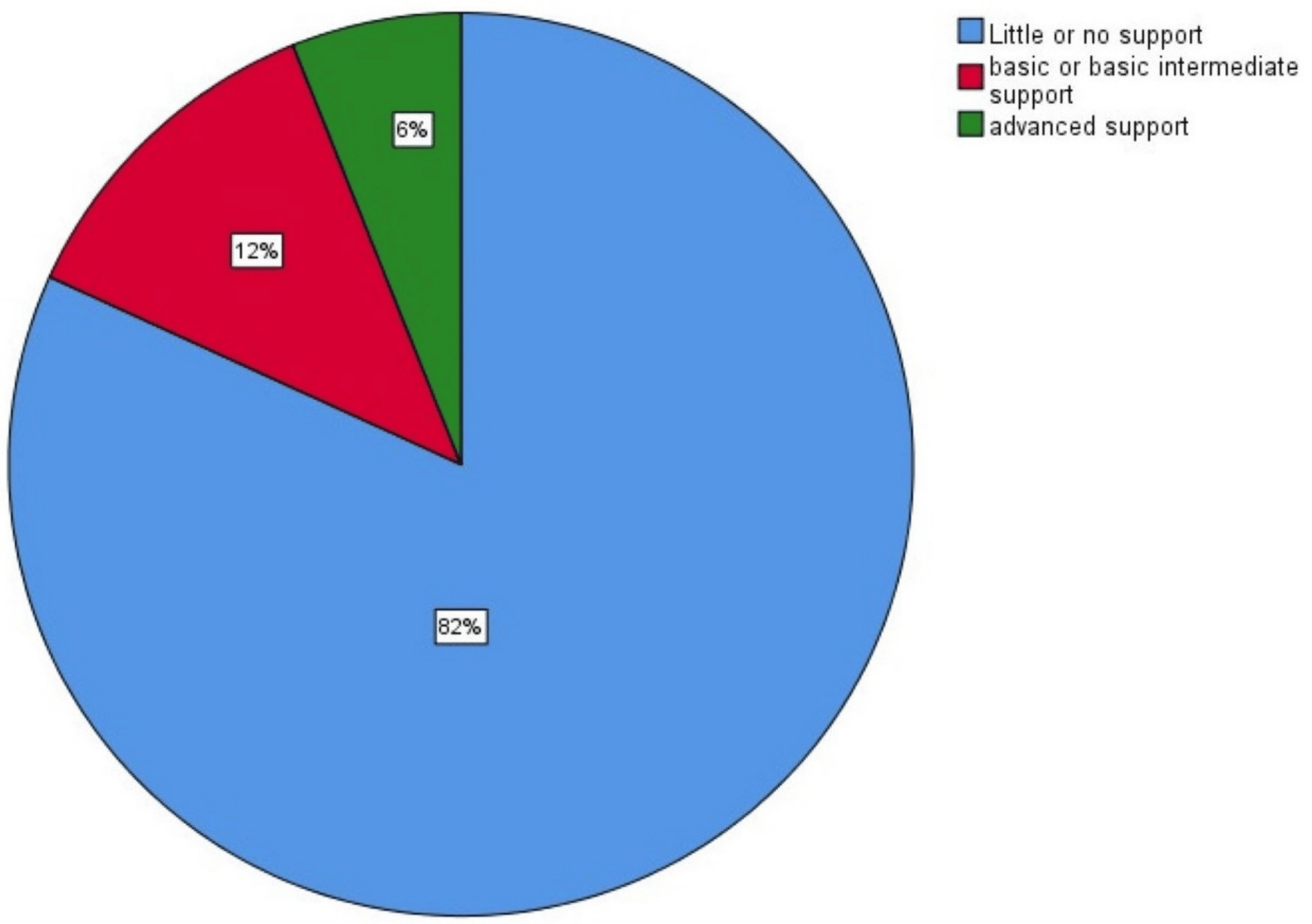
A pie chart showing overall support for MMI health care in maternity care settings MMI: maternal mental illness

This was obtained by health workers scoring on the seven individual components of the ACIC tool according to how they viewed performance in their respective health facilities regarding MMH care. The total scores for the individual parameters under that component divided by their number of items gave the sub-average scores for the respective components, while the overall average was obtained by the sum of the scores for the seven components divided by the total number of the components as shown in Table 2 and Figure 2 below.

**Table 2:** Maternal care providers’ rating of capacity of health workers and facilities to manage MMI using the ACIC tool ACIC: Assessment of Chronic Illness Care; MMI: maternal mental illness Average scores are reported for the respective components of the ACIC tool

ACIC tool component, n=33	Frequency (percentage)
Organization of the healthcare delivery system	
0-2 (little or no support)	26 (78.8)
3-5 (basic support)	6 (18.2)
6-8 (advanced support)	1 (3.0)
9-11 (optimal support)	0 (0.0)
Community linkages	
Little or no support	26 (78.8)
Basic support	5 (15.2)
Optimal support	1 (3.0)
Advanced support	1 (3.0)
Self-management support	
Little or no support	22 (66.7)
Basic support	6 (18.2)
Advanced support	5 (15.2)
Optimal support	0 (0.0)
Decision support	
Little or no support	23 (69.7)
Basic support	9 (27.3)
Advanced support	0 (0.0)
Optimal support	1 (3.0)
Delivery system design	
Little or no support	24 (72.7)
Basic support	5 (15.2)
Advanced support	3 (9.1)
Optimal support	1 (3.0)
Clinical information systems	
Little or no support	24 (72.7)
Basic support	8 (24.2)
Advanced support	1 (3.0)
Optimal support	0 (0.0)
Integration of chronic care components	
Little or no support	26 (78.8)
Basic support	6 (18.2)
Advanced support	1 (3.0)
Optimal support	0 (0.0)

Results in Table 2 revealed little or no support for MMI care in maternity care settings based on most of the components assessed and none on optimal support.

Overall, 81.8% scored between 0 and 2 on the ACIC tool components, which correspond to little or no support for MMH illness care in primary and tertiary settings, as shown in Figure 2.

Chart review findings for women screened positive for maternal mental health problems

**Figure 3:**
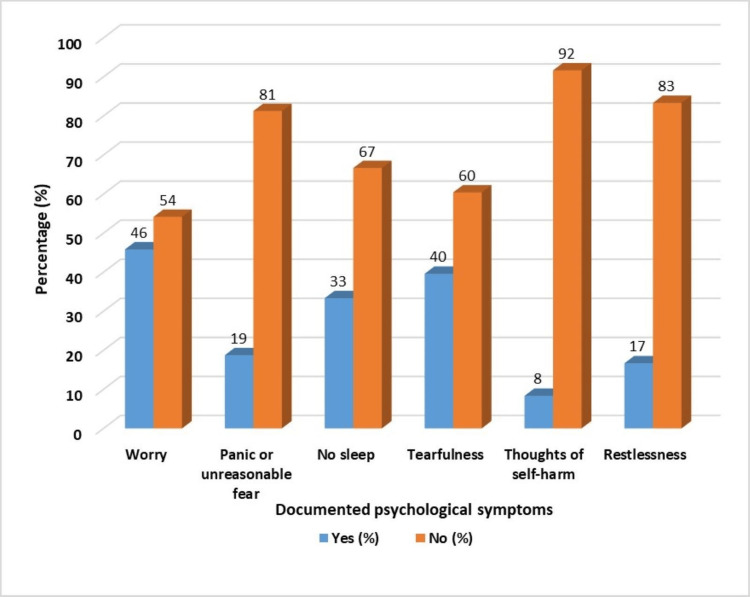
A bar graph showing documented record of psychological symptoms assessed and documented by health workers among women with known maternal mental illness symptoms %: percentages

The findings from the review of the case records of participants with known mental illness symptoms after being reviewed by maternal care providers are shown in Figure 3.

From Figure 3, the detection of psychological symptoms related to mental illness among women attending maternity care services was average for most symptoms except the symptom of worry which was assessed for about 46%.

From Table 3, the majority of participants (66.7%) who screened positive for MMI shortly before being reviewed by maternal care providers were not detected as having a psychological problem by the facility's maternal care providers (p=0.001). Women with documented psychological symptoms had higher chances of receiving psychological treatment (p=0.006).

**Table 3:** Level of detection and management of women screened positive for MMI prior to being seen by maternal care providers The test statistic reported for p-values was the chi-square test, but Fisher's exact test was reported for more than 20% of expected cell counts with less than 5. P-values corresponding to statistically significant values are denoted by an asterisk (*).

	Documented diagnosis			Psychological treatment			Pharmacological treatment		
	No, n (%)	Yes, n (%)	p-value	No, n (%)	Yes, n (%)	p-value	No, n (%)	Yes, n (%)	p-value
Psychological symptoms	NA	NA	0.000*	NA	NA	0.006*	NA	NA	0.111
No, n=23	23 (47.9)	0	NA	20 (41.7)	3 (6.3)	NA	23 (47.9)	0	NA
Yes, n=25	9 (18.8)	16 (33.3)	NA	12 (25.0)	13 (27.1)	NA	21 (43.8)	4 (8.3)	NA

As per Table 4, most participants reported more aspects of accessibility but less of patient abilities. The majority of participants (≥68%) reported that maternal care providers were approachable, welcoming, and available and that healthcare services were available and appropriate. More than half (52.1%) of the women who screened positive for MMI did not consider themselves to be sick, and more than half (54.2%) lacked the financial ability to pay for medical care.

**Table 4:** Participants’ rating of maternal mental care access and abilities

Variables	Yes (%)	No (%)
Accessibility of health care services	-	-
Health workers were approachable	35 (72.9)	13 (27.1)
Felt accepted/welcomed by the health worker	37 (77.1)	11 (22.9)
Health workers were available and accommodated us	38 (79.2)	10 (20.8)
Services were available at the facility	36 (75.0)	12 (25.0)
Received appropriate interventions	33 (68.8)	15 (31.2)
Patient abilities to access health care	-	-
Whether woman perceived that she was sick	23 (47.9)	25 (52.1)
Able to seek for care	26 (54.2)	22 (45.8)
Able to reach out for care	29 (60.4)	19 (39.6)
Able to pay for the care	22 (45.8)	26 (54.2)
Able to engage in health care services	30 (62.0)	18 (37.5)

Women’s perceptions of mental health services meeting their expectations and needs

In-depth qualitative interviews were carried out among women with MMI to explore their perceptions of mental health services with respect to meeting their expectations and care needs. Three complementary themes were identified: i) met and unmet service expectations; ii) concerns about healthcare providers’ skills and knowledge; and iii) barriers to mental healthcare services use.

Theme One: Met and Unmet Service Expectations

Respondents identified a wide variety of expectations and the degree to which they were met at healthcare institutions. Many participants anticipated health workers to spend time talking with them and analyzing whether they have any health concerns regarding the pregnancy or their overall health. They expect health workers to give them relevant information and advice about healthy living. The following representative quote exemplified these expectations.

 “They should add more on assessing what is wrong with us in addition to checking the abdomen. They should counsel and give you hope.” (024 Woman Positive, Antenatal)

“….sometimes you come, you sit for long, [and] no one is talking to you. And yet when they talk to you, you also remain firm…” (001 Woman Positive, Antenatal).

“…They counselled, and they have taught me what to do and about the sickness. I feel I'm satisfied with the care… (010 Woman Positive, postnatal).

Theme Two: Concerns About Healthcare Providers’ Skills and Knowledge

Respondents were concerned about the poor communication skills of some maternal care providers. A number of respondents reported that some healthcare workers communicated inappropriately by yelling at the mothers, making them more distressed. Some respondents questioned the competence of certain health workers to assess and manage maternal mental health issues. The following representative quotes illustrated these concerns:

“… They shouldn’t be talking badly because they add on stress to stress. They should ask women to know their problems and counsel them…” (019 Woman Positive, Antenatal).

"…Train health workers to assess for stress and mental issues. You find the health workers we meet don’t know about these [mental] problems we are facing. So they can’t ask about them..." (009 Woman Positive, Postnatal).

Theme Three: Barriers to Mental Care Services Access and Utilization by Perinatal Women

Perinatal women identified a number of barriers to accessing and using mental health care services within maternity care, including medicine stockouts, a small number of health workers responding to a large number of patients resulting in lengthy waiting periods, failure of women with MMI to recognize the need for care, and a lack of funds to purchase drugs, among others. These concerns are illustrated by the following quotes:

“…They only examine the abdomen and give the return date. The medicine for the pregnancy, they give but sometimes you are asked to buy it, and you don’t have the money” (024, Woman Positive, Antenatal).

“ …I haven’t sought for any help regarding my issues (mental illness symptoms) because I know I haven’t got any mental breakdown so far.” (009 Woman Positive, postnatal).

“…even health workers were few, we were many mothers all waiting for one nurse (001 Woman Positive, Antenatal).

## Discussion

In our study, 81.8% of maternal care providers reported little or no support for maternal mental health care. The study further demonstrated a low level of detection of MMIs, where only 33.3% of women with maternal depression and anxiety were detected by maternal care providers. Furthermore, only a small minority of those diagnosed obtained appropriate treatment (27.1% psychological and 8.3% pharmacological). The majority of respondents (≥68%) reported that maternal care providers were approachable, welcoming, and available and that healthcare services were available and appropriate. However, more than half (52.1%) of the women who screened positive for MMI did not consider themselves to be sick, and more than half (54.2%) lacked the financial ability to pay for medical care. A shortage of maternal care workers and poor communication, assessment, and management skills among health practitioners in maternity care settings were identified as barriers to accessing maternal mental health services.

Our study found that healthcare workers and facilities had a very low capacity to identify and treat MMIs, which was consistent with a number of previous studies in other parts of the world [1,9,24]. In contrast, Harrison et al. revealed that a significant percentage of perinatal women were evaluated for mental health both prenatally (83.4%) and postnatally (73.7%) [25]. However, the Harrison study's greater sample size and longer length/duration of study (2014-2020) may be the cause of this disparity. Furthermore, the Harrison study was also carried out in the United Kingdom, a nation with a more advanced healthcare system, where the government mandated maternity care providers to evaluate each perinatal mother's mental health during the time of data collection. Nevertheless, a number of studies have shown that although routine mental health screening increases the proportion of mothers who are assessed for mental illness, it has no appreciable impact on the initiation and preference of pharmacological or psychological therapy for those women who receive a diagnosis [26]. This is most likely the result of the maternal care providers' lack of appropriate training.

Maternal care providers lack the skills and knowledge necessary to effectively assess and diagnose MMI since midwives in particular and other maternal care specialists in general do not receive special training on maternal mental health [27,28]. There is a need to teach appropriate skills and knowledge to non-specialist mental care providers to treat MMI to reduce the high burden of undetected and untreated mental health illnesses. There is a growing body of research evidence suggesting enhanced communication skills, empathy, and MMI detection and management in areas where non-specialized community health workers were taught to screen and manage mental illness [15,29]. Thus, implementing task-shifting mhGAP interventions in maternity care services, which include educating maternal care providers on how to diagnose and manage MMI, could be a potential strategy for resolving Uganda's low MMI detection and management rates.

In our study, a substantial proportion of women with MMI (52.1%) did not realize they were sick, and nearly similar proportions (54.2%) were unable to afford mental healthcare services. These findings, combined with women with MMI rating maternal mental health care services to be outstanding despite the low level of MMI detection and management within the same cohort, demonstrate personal barriers to quality MMH care. These are critical impediments to accessing good maternal mental care by women with MMI in the study setting. However, numerous previous studies in other settings reported similar findings of women with MMI who are unable to recognize that they are sick [26,30], unable to pay for mental health care [30], and perceive inadequate maternal mental health care services to be excellent [1]. Certain socio-cultural views, such as viewing signs and symptoms of prenatal mental illness as a reflection of a weak character [26], maybe the cause for symptomatic women to disregard MMI symptoms and avoid obtaining medical care. High levels of poverty in rural settings, as well as ignorance of what constitutes quality healthcare, could be the reason why many respondents could not afford to pay for care services and failed to detect substandard care services, respectively. Consequently, stakeholders in maternal mental care may need to provide culturally tailored awareness campaigns to educate the public about the signs, symptoms, and management of MMI to promote early detection and treatment for symptomatic mothers. Furthermore, there is a need to ensure adequate stocking of appropriate drugs for MMI as a short-term intervention to drug outages and to adopt policies that economically empower women, such as equitable access to education and jobs as a long-term solution to inaccessibility to quality maternal care services. 

Strengths and limitations of the study

The small sample size for the quantitative findings for health personnel and women who tested positive for MMI may limit the generalizability of the results. However, the triangulation of collection methods and participant groups improved the data's generalizability. Furthermore, data was collected in a number of settings, ranging from health center level III to referral level, prenatal and postnatal settings, including the only two regional referral hospitals in the region, assuring representation. Finally, women were recruited during perinatal visits, and interviews were conducted in health centers. This may have motivated participants to provide responses deemed desirable by healthcare providers. However, we promoted confidentiality during data collection to avoid undue influence on participants.

## Conclusions

This study demonstrated a low capacity to detect MMI in maternity care settings; maternal care providers detect and treat women with MMI at very low rates. A substantial number of women with MMI are unable to realize that they are sick and require mental health assistance. The findings highlight the need for research to enhance recognition and treatment for symptomatic mothers and their families and the general population to enable them to realize the need to access and utilize MMI services, hence improving the quality of care.

## Appendices

Health workers’ questionnaire on assessment of capacity to care for MMH problems in the maternity care settings

Appendix A

A. Sociodemographic characteristics of health workers

**Table 5:** Health workers' sociodemographic characteristics

A.	Sociodemographic characteristics of health workers		
S/N	Variable	Description	Tick applicable response
1.	Health facility level	Health Center III	
		Health Center IV	
		Referral facility	
2.	Department	Antenatal clinic	
		Postnatal	
		Immunization clinic	
		Other, specify, ……….………	
3.	Position held	Unit in-charge	
		Frontline healthcare provider	
4	Designation	Nurse	
		Midwife	
		Doctor/Obstetrician	
		Other	
5	Qualifications	Certificate	
		Diploma	
		Degree	
6	Gender	Female	
		Male	
7	Age (years)		…………………
8	Years of experience in the maternity setting		………………….

Appendix B

B. Please rate the degree to which the following descriptions apply to the capacity of your facility to care for women with mental health problems during pregnancy and after delivery (The range is 0-11; whereby: 0-2 (little or no support for MMH illness care); 3-5 (basic or intermediate support for chronic illness care); 6-8 advanced support); and 9-11 (optimal, or comprehensive integrated MMH care). Write the number corresponding to the rating scored for the respective components

**Table 6:** Assessment of the capacity to detect and manage MMI in maternity care settings using the ACIC tool HCIII: Health Centre III; HCIV: Health Centre IV; ACIC: Assessment of Chronic Illness Care; MMI: maternal mental illness; MMH: maternal mental health

S/N	ACIC tool components	Score
A	Organization of the Healthcare Delivery System	
1.	Overall organizational leadership in MMH care	
2.	Organizational goals for MMH care	
3.	Improvement strategy of MMH care	
4.	Incentives and regulations in MMH care	
5.	Senior leaders responsible for MMH care	
6.	Benefits	
	Sub Average (Total score/number of items)	
B	Community linkages	
7.	Linking patients with MMH problems to outside resources	
8.	Partnership with community organizations	
9.	Regional health plans for MMH care	
	Sub Average (Total score/number of items)	
C.	Self-management support	
10.	Assessment and documentation of self-management needs and activities	
11.	Self-management support to clients with MMH problems	
12.	Addressing concerns of patients and families affected	
13.	Effective behavior changes interventions and peer support for the affected	
	Sub Average (Total score/number of items)	
D.	Decision support	
14.	Evidence-based guidelines in line with MMH care	
15.	Involvement of specialists in improving primary care for MMH problems	
16.	Providing education on MMH problems and care	
17.	Informing patients about guidelines available	
	Sub Average (Total/number)	
E.	Delivery system design	
18.	Practice team functioning to deliver MMH care	
19.	Practice team leadership to deliver MMH care	
20.	Appointment system for the patients with MMH problems	
21.	Follow-up for the patients with MMH problems	
22.	Planned visits for patients/women with MMH problems	
23.	Continuity of care for women with MMH problems	
	Sub Average (Total/number of items)	
F	Clinical information systems	
24.	Registry (list of patients with specific conditions)	
25.	Reminders to providers on screening and management of MMH problems	
26.	Feedback from women and health frontline health care workers	
27.	Information about relevant subgroups of patients needing service	
28.	Patient treatment plans for the patients/women	
	Sub Average (Total score/number of items)	
G	Integration of chronic care components	
29.	Informing patients about guidelines	
30.	Information systems/registries	
31.	Community programs	
32.	Organizational planning for MMH care	
33.	Routine follow-up for appointments patient assessments and goal planning	
34.	Guidelines for MMH care	
	Sub Average (Total score/number of items)	
	OVERALL AVERAGE	

Questionnaire for antepartum and postpartum women screened positive for maternal mental illness regarding interventions for detecting and managing MMH problems from the maternity care setting and rating their met and unmet mental health needs

Appendix C

**Table 7:** Chart records review for mothers seen by the facility health care providers

S/N	Item	Participants’ response	Tick what applies	
1.	Health facility	Health Center III		
		Health Centre IV		
		Referral facility		
2.	Department	Antenatal clinic		
		Postnatal		
		Immunization clinic		
		Other, specify, ……….………		
	Please tick appropriately for Yes or No		Yes	No
3	Psychological symptoms recorded	Worry		
		Tearfulness		
		No appetite		
		No sleep		
		Restlessness		
		Fixed beliefs centered around the baby		
		Panic or unreasonable fear		
		Attempts of self-harm		
		Attempts of harming baby		
		Others, specify;		
4.	Clinical impression or diagnosis given	……………………………………		
5	Record of psychological treatment	Counselling		
		Psychotherapy		
		Family emotional support		
		Others, specify; …..………………		
6	Record of pharmacological interventions	Antidepressants		
		Anti-anxiety drugs		
		Other, specify, …………………		
7	Follow up care	Return date given		
		Referral to community health care		
		Referral to specialist care		
		Other, specify; …………………		

Appendix D

**Table 8:** Women’s rating of quality of care received and accessibility of interventions for MMI care in maternity care settings HCIII: Health Centre III; HCIV: Health Centre IV; MMI: maternal mental illness

S/N	Item	Yes	No
	Overall quality of care provided in the clinics		
	Are you satisfied with the care provided at the facility?		
	Have you had any difficulty in accessing health care services?		
	Did you ever feel embarrassed or ashamed of seeking treatment at the health facility?		
	Accessibility of health workers at the facility		
	Were the health workers approachable?		
	Did you feel accepted or welcomed by the health workers?		
	Were they available and able to accommodate you as a patient and your family care givers?		
	Were the services affordable?		
	Did you receive appropriate interventions to your health condition?		
	Patient abilities to access health care		
	Did you perceive that you were at risk or that you were sick?		
	Were you able to seek for care?		
	Were you able to reach out for the care?		
	Were you able to pay for the care?		
	Were you able to engage in health care services successfully?		

Appendix E

Section C. Needs and support for women and their family care givers during MMH care access

1. Kindly tell me the kinds of health care needs or support you expect health care workers in the maternity care settings to meet in care of the maternal mental problems.

i) For the women

ii) For the family care givers

2. What needs were not met by the health care providers in the maternity care settings for the MMH problems?

i) For the woman

ii) For the family care givers

3. What are the challenges encountered to access MMH care?

4. Please share any other information you would like to regarding MMI care
